# Drug Combination Nanoparticles Containing Gemcitabine and Paclitaxel Enable Orthotopic 4T1 Breast Tumor Regression

**DOI:** 10.3390/cancers16162792

**Published:** 2024-08-08

**Authors:** Jesse Yu, Xiaolin Xu, James Ian Griffin, Qingxin Mu, Rodney J. Y. Ho

**Affiliations:** 1 Department of Pharmaceutics, University of Washington, Seattle, WA 98195, USA; yuj73@uw.edu (J.Y.); alinxu@uw.edu (X.X.);; 2 Department of Bioengineering, University of Washington, Seattle, WA 98195, USA

**Keywords:** gemcitabine, paclitaxel, drug-combination, nanoparticles, breast cancer, lymphatic delivery, synchronized delivery, target localization

## Abstract

**Simple Summary:**

Breast cancer typically originates and metastasizes from the mammary fat pad. The tumor within the fat pad is supported by enriched lymphatic vessels, to a greater degree than that of blood vessels. Recently, we have successfully co-formulated two physically disparate cancer drugs, water-soluble gemcitabine (G) and water-insoluble paclitaxel (T), into a drug combination nanoparticle (DcNP) referred to as GT-in-DcNP. This GT-in-DcNP dosage form is stable and scalable. When it is given to mice, it enables synchronized delivery of GT into tumors via enriched lymph vessels. A single dose of GT-in-DcNP in mice has been found to suppress breast tumor growth in the mammary fat pad more effectively than an equivalent dose of the free GT combination. We observed tumor regression and restoration of mammary fat tissue at higher doses of GT-in-DcNP. This concept has been extended to demonstrate the ability to suppress human breast cancer in a xenograft model. With these observations, GT-in-DcNP may be considered for clinical development to treat breast cancer.

**Abstract:**

Early diagnosis, intervention, and therapeutic advancements have extended the lives of breast cancer patients; however, even with molecularly targeted therapies, many patients eventually progress to metastatic cancer. Recent data suggest that residual breast cancer cells often reside in the lymphatic system before rapidly spreading through the bloodstream. To address this challenge, an effective drug combination composed of gemcitabine (G) and paclitaxel (T) is administered intravenously in sequence at the metastatic stage, but intravenous GT infusion may limit lymphatic GT drug accessibility and asynchronous drug exposure in cancer cells within the lymph. To determine whether co-localization of intracellular gemcitabine and paclitaxel (referred to as GT) could overcome these limitations and enhance the efficacy of GT, we have evaluated a previously reported GT drug-combination formulated in nanoparticle (referred to as GT-in-DcNP) evaluated in an orthotopic breast tumor model. Previously, with indocyanine green-labeled nanoparticles, we reported that GT-in-DcNP particles after subcutaneous dosing were taken up rapidly and preferentially into the lymph instead of blood vessels. The pharmacokinetic study showed enhanced co-localization of GT within the tumors and likely through lymphatic access, before drug apparency in the plasma leading to apparent long-acting plasma time-course. The mechanisms may be related to significantly greater inhibitions of tumor growth—by 100 to 140 times—in both sub-iliac and axillary regions compared to the equivalent dosing with free-and-soluble GT formulation. Furthermore, GT-in-DcNP exhibited dose-dependent effects with significant tumor regression. In contrast, even at the highest dose of free GT combination, only a modest tumor growth reduction was notable. Preliminary studies with MDA-231-HM human breast cancer in an orthotopic xenograft model indicated that GT-in-DcNP may be effective in suppressing human breast tumor growth. Taken together, the synchronized delivery of GT-in-DcNP to mammary tumors through the lymphatic system offers enhanced cellular retention and greater efficacy.

## 1. Introduction

Early-stage breast cancer patients are typically treated through surgical resection of the primary tumor, radiation therapy, and combinations of oral or systemic drug therapies based on the specific characteristics of the breast cancer (i.e., HER^2+^, ER/PR^+^, triple negative for the mentioned receptors/markers, etc.) [1]. This multimodal and interventional approach is effective in keeping over 90% of patients in remission for up to 5 years after initial diagnosis. Unfortunately, ~30% of patients who may respond to current therapy may eventually progress to metastatic disease that leads to poor prognosis [2,3]. Recently, two independent metastatic cell migration studies indicated that breast cancer 4T1 cells can traverse to the draining lymph nodes and subsequently spread to distant nodes throughout the lymphatics. This process eventually leads to an invasion of cancer cells into the blood vessels [4,5]. Once in the blood, cancer cells can proliferate rapidly and spread to distal tissues, likely due to high blood perfusion (flow) rates. Breast cancer cells carried by the systemic circulation can aggregate and localize in blood capillaries in the lung as metastatic nodules. Thus, tumor nodules in the lungs are considered a feature of late-stage metastatic breast cancer. On the other hand, these studies also point to the slow-growing rates of breast cancer cells initially found in the lymph that may reflect early-stage metastasis in the lymph through the lymph nodes. Without complete clearance, residual cells in lymphatics may drive metastatic disease progression, likely through spreading into the systemic circulation via higher blood flow and perfusion to major organs. 

In contrast to oral medications, intravenously administered therapeutics can directly access systemic circulation without exposure to first-pass metabolism. Drugs given intravenously (IV) can directly access tumor tissues through blood but are also subjected to competing processes such as metabolic inactivation, distribution into peripheral tissues, and excretory clearance. Additionally, these molecules are subject to blood-to-lymph barriers before gaining access to the lymphatic tissues where cancer cells may also reside [6]. Thus, lymphatic drug concentration and overall exposure are likely lower than that in blood through current oral or IV dosing of cancer therapeutics. Currently, the treatment strategies with oral or IV dosage forms for breast cancer are focused on systemic exposure and thus may be sub-optimal for eliminating cancer cells in the lymph [6]. Limited overall lymphatic drug exposure with current chemotherapeutic dosing regimens may relate to limited clearance of breast cancer cells in the lymph and a progression to metastasis. 

Over the course of breast cancer treatment, molecular targeted therapies such as kinase inhibitors or hormone receptor antagonists are used earlier in treatment regimens based on their more tolerable toxicity profiles [7]. However, these targeted therapies are known to develop drug resistance, particularly in single-drug treatment regimens [8]. When tumors no longer respond to targeted therapies, broad-acting drugs, often referred to as chemotherapy, are used. Unfortunately, due to the dose-limiting toxicities and non-specific mechanisms of action in chemotherapy, intolerable side-effects often pose a significant challenge for achieving cancer remission. Chemotherapy is typically administered intravenously as a multiple-drug combination regimen to maximize tumor killing and delay disease progression. While a number of chemotherapeutic agents are recommended in national guidelines to treat breast cancer [9], many agents exhibit dose-limiting toxicities. For example, anthracycline-based agents such as doxorubicin and daunorubicin can cause cumulative cardiotoxicity due to their irreversible binding to cardiolipin [10], and those drugs have lifetime dose restrictions. A taxane-based combination referred to as gemcitabine (G) and paclitaxel (T) or GT is used extensively for metastatic breast cancer treatment and is a non-anthracycline alternative. Unlike anthracycline derivatives, GT treatment does not carry a lifetime dose limitation [11,12]. GT kills cancer cells by targeting DNA synthesis (G) and tubulin disassembly (T) [13]. If one can synchronize the presence of both GT together in a single cancer cell over an extended period of time, it may reduce the risk of drug resistance and potentially provide sustained suppression of cancer cell growth and metastatic disease progression. Thus, the combined delivery of GT in one entity may improve the effectiveness of the treatment regimen. 

To bring together water-insoluble paclitaxel and water-soluble gemcitabine in a nanoparticulate form, we have previously reported success in the creation of a stable GT drug combination in novel nanoparticles referred to as GT-in-DcNP [14,15]. We found that when given GT-in-DcNP by IV route, co-delivery as a nanoparticle enhances the effects of GT compared to a freely solubilized control and, at an optimal dose, completely clears metastatic breast cancer nodules in the lungs [14,15,16]. Additional drug disposition studies suggest that the enhancement of GT-in-DcNP therapeutic effects is likely due to the extended plasma time-course of both gemcitabine and paclitaxel in the blood after IV dosing [15]. Compared to free-soluble drug treatment, stabilized drug molecules that are formulated in nanoparticles may potentially enhance circulation time and improve the uptake and retention of G and T in breast cancer cells colonizing the lung. As metastatic breast cancer nodules in the lung are considered a late-stage feature of the disease, a treatment strategy that can address the early spread of cancer growth (e.g., in the lymph) could prevent or delay disease progression. 

In this study, we have evaluated whether subcutaneous GT-in-DcNP can enhance synchronized GT delivery to 4T1 breast cancer in mammary fat-pads, potentially through distribution and retention of GT-in-DcNP from subcutaneous to lymph vessels and the lymphatic systems, including retention in the nodes. For this purpose, we first developed and characterized an orthotopic model of early-stage breast cancer to test this hypothesis. Subsequently, this orthotopic 4T1 breast tumor model is used to evaluate the lymphatic accumulation of GT-in-DcNP or an equivalent free-soluble form administered through subcutaneous injection. We found that the GT-in-DcNP was first taken up and retained in the lymph vessels and nodes. Compared to soluble drug formulation controls, a single subcutaneous dose of GT-in-DcNP has led to tumor regression, as measured by the in vivo imaging system (IVIS). This may be related to synchronized GT uptake and retention in the tumor-laden tissues.

## 2. Materials and Methods

### 2.1. Materials

Gemcitabine (G) free base (>99%) and paclitaxel (T) (>99.5%) were purchased from LC Laboratories (Woburn, MA, USA). GMP grade lipid excipients—1,2-distearoyl-sn-glycero-3-phosphocholine (DSPC) and N-(carbonylmethoxypolyethyleneglycol with MW = 2000)-1,2-distearoyl-sn-glycero-3-phosphoethanolamine sodium salt (DSPE-mPEG_2000_) was purchased from Cordon Pharma (Liestal, Switzerland). D-Luciferin was purchased from Perkin Elmer (Waltham, MA, USA). Indocyanine green (ICG) was purchased from Sigma-Aldrich (St. Louis, MO, USA). Anhydrous ethanol was purchased from Decon Pharmaceuticals (King of Prussia, PA, USA). Dimethyl sulfoxide (DMSO) was purchased from Fisher Chemical (Fair Lawn, NJ, USA). All chemical reagents were of analytical grade or higher. Isoflurane was purchased from Piramal Critical Care, Inc. (Bethlehem, PA, USA). The metastatic breast cancer 4T1 cell line stably transfected with luciferase and green fluorescence protein (GFP) plasmid (referred to as 4T1-luc) was kindly provided by the Stanley Riddell laboratory at the Fred Hutchinson Cancer Center (Seattle, WA, USA). MDA-231-HM, a brain-metastatic derivative of MDA-MB-231 transfected with luciferase and GFP, was a gift from City of Hope (Duarte, CA, USA) [17]. Luciferase and GFP expressions were validated before performing experiments. 

### 2.2. Preparation and Characterization of Gemcitabine and Paclitaxel (GT) in Drug Combination Nanoparticles

**Scheme 1:**
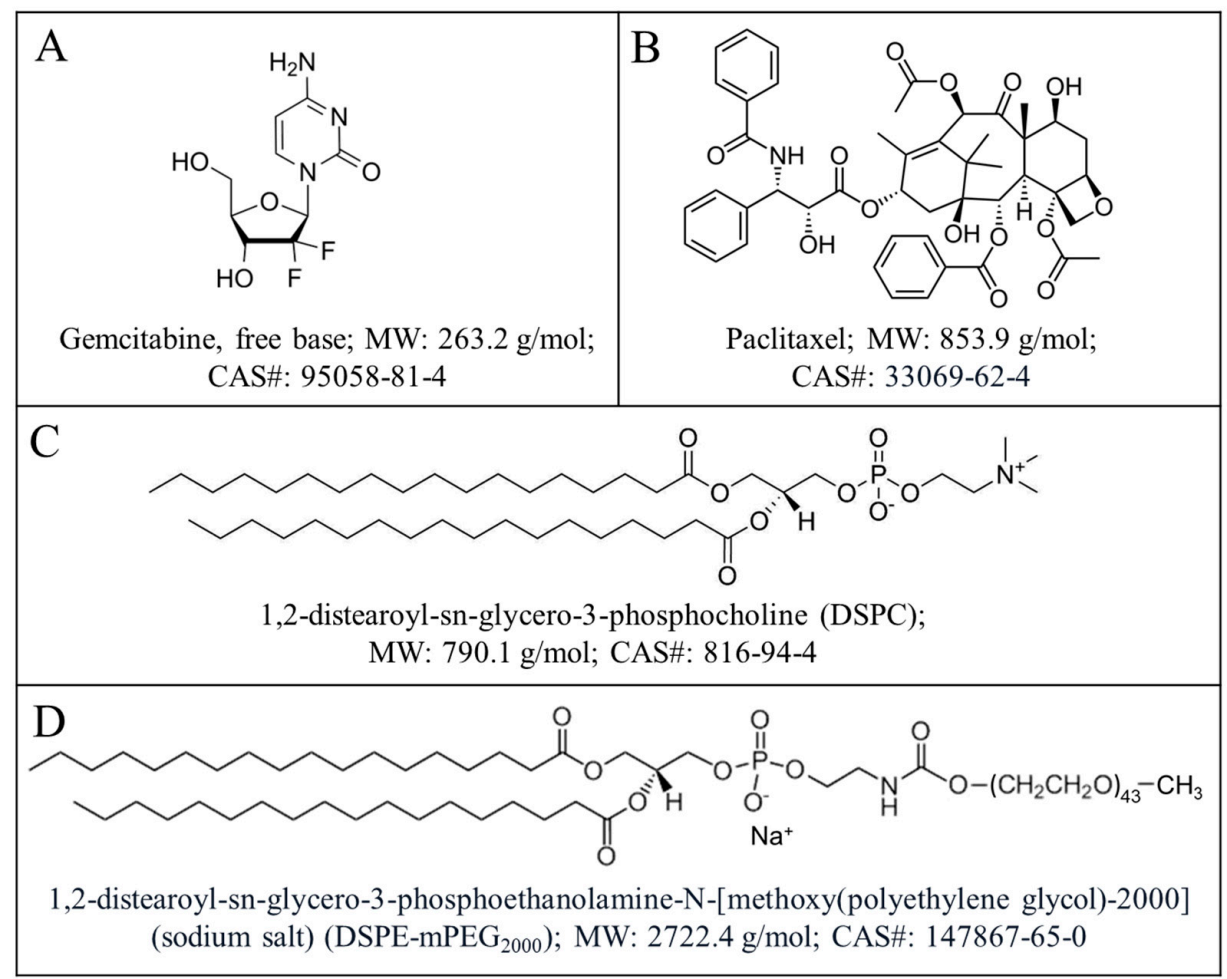
Chemical structures, full names, molecular weights (MW), and CAS registry numbers of drugs: gemcitabine (**A**), paclitaxel (**B**), and lipid excipients: DSPC (**C**) and DSPE-mPEG_2000_ (**D**) of the GT-in-DcNP formulation.

Drug combination nanoparticles (DcNP) composed of gemcitabine and paclitaxel at a fixed weight ratio of 10:1 (*w*/*w*) and referred to as GT-in-DcNP were prepared as previously described (Scheme 1) [15]. Briefly, 7 mg of gemcitabine (G) and 0.7 mg of paclitaxel (T) were dissolved in a flask in ethanol (60 °C); then 25 mg of DSPC and 10 mg of DSPE-mPEG_2000_ were added for each mL of drug and lipid solution. The final concentration of solutes (GT drugs + DSPC/DSPE-mPEG_2000_ lipid excipients) in ethanol was 5% *w*/*v*. The solvent was removed under controlled rotary evaporation followed by vacuum desiccation to achieve dryness overnight at room temperature. The dried GT-in-DcNP powder product was hydrated in 0.45% NaCl with 20 mM NaHCO_3_ buffer at 70 °C, with pH 7.4, to achieve a final lipid concentration of 100 mM. The GT-in-DcNP in suspension was subjected to size-reduction in a bath-type sonicator (Avanti Polar Lipids, Inc., Alabaster, AL, USA) (5 min on, 5 min off, 3 cycles) to obtain stable GT-in-DcNP nanoparticles in suspension. The particle size of GT-in-DcNP was determined with a dynamic light scattering (DLS) particle analyzer, NICOMP 380 ZLS (Particle Sizing Systems, Santa Barbara, CA, USA).

To track GT-in-DcNP in mice, a near-infrared tracer ICG 0.4% [ICG: (DSPC + DSPE-mPEG_2000_) = 1:250] was added in the GT-in-DcNP preparation. Briefly, ICG was first dissolved in methanol prior to mixing with the ethanol solution containing drugs and lipids as described above. Solvent removal, hydration, and particle size reduction were performed as described for GT-in-DcNP preparation, but the process was performed in the dark to prevent light-dependent ICG degradation [16].

### 2.3. Preparation of Freely and Soluble Gemcitabine and Paclitaxel (GT) Combination

To prepare an equivalent free and soluble GT drug combination for use as a control, G and T were dissolved in DMSO as a stock solution (G: 50 mg/mL, T: 5 mg/mL), followed by 10-fold or more dilution with normal saline; such that final DMSO content is 10% less (*v*/*v*) or lower. The drug combination in DMSO was used immediately as a free-soluble drug-combination control treatment in animal studies.

### 2.4. Development and Characterization of 4T1 Breast Tumor in Mouse Mammary Fat Pads

**Figure 1:**
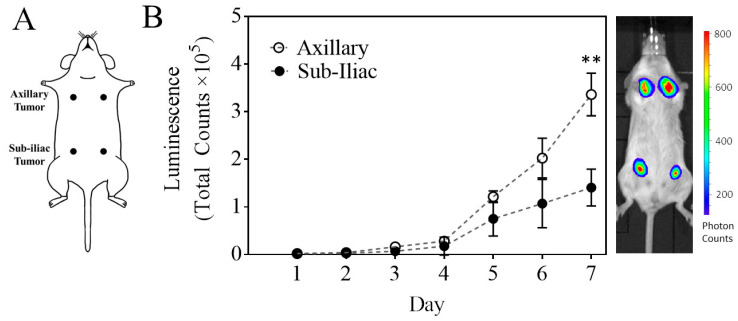
Characterization of a primary multi-site 4T1-based model of breast cancer. Mice were inoculated with 1 million 4T1-luc cells at four different sites (Panel (**A**)). Tumors located near the axillary fat pads exhibited statistically distinct growth patterns compared to tumors located near the sub-iliac fat pads (Panel (**B**)) as determined by luminescent measurements over time (mean ± SEM). ** *p* < 0.001, Student’s *t*-test. On the right of Panel (**B**) is a representative bioluminescence image of a mouse with 4T1 tumors on day 7.

All animal studies were conducted with an Institute of Animal Care and Use Committee (IACUC) approved protocol at the University of Washington. All studies involving mice in this report are based on statistical designs to ensure that enough animals in each group are enrolled to discern differences between test and control groups. As breast tumor incidents predominantly occur in females, this report focuses on female mice, and we intend to study the role of sex as a variable when GT-in-DcNP progresses to a preclinical drug candidate stage. Female BALB/c mice (6–8 weeks old) were purchased from The Jackson Laboratory (Bar Harbor, Maine) and housed in an animal research facility for at least one week before use. Metastatic breast cancer 4T1-luc cells were first verified in vitro to express luciferase (luc) and GFP (green fluorescence protein) to enable monitoring of cancer cell growth via bioluminescence. 4T1-luc cells (0.2, 0.5, or 1 million cells) were detached into single-cell suspensions. Cells were suspended in 50 µL of ice-cold Hank’s balanced salt solution (HBSS) at different concentrations and were subcutaneously inoculated under anesthesia in the left and right mammary fat pads adjacent to the axillary and sub iliac lymph nodes (underneath nipples #1,4,6,9) (Figure 1A. Injections were mirrored across the midline of the mouse. Multiple cell inoculations on singular mice were used to facilitate more robust data and reduce the total number of animals needed for the study. Mice were monitored daily for the duration of the study. Bioluminescence of 4T1-luc breast cancer cells in the mice was examined with a XENOGEN IVIS 200 imaging system (PerkinElmer, Inc., Waltham, MA, USA). To measure the bioluminescence of 4T1 cells in vivo, the mice were first given 150 mg/kg D-luciferin by intraperitoneal injections, and imaging was performed within 10~15 min. The XENOGEN IVIS imaging parameters were as follows: field of view, 12; excitation filter, closed; emission filter, open; exposure time, 120 s; binning factor, 4; f/stop, 2. A total of 4T1-luc bioluminescence emission from the mice was integrated using Live Image software, version 4.7.3 (PerkinElmer, Waltham, MA, USA). Luminescence was used as an indicator for cancer growth from Day 0–3. Once tumors were palpable, caliper measurements were taken to corroborate the luminescent signals. 

### 2.5. Immunohistochemistry (IHC) Staining of CD31 and Podoplanin to Detect Developing Tumor Blood and Lymphatic Vasculature

Tumor tissues were collected 5 days after mice were inoculated subcutaneously with 1 million cells each in the axillary and sub-iliac regions. Tissues were fixed in 10% formalin and stored in 70% ethanol before being embedded into paraffin blocks. CD31 and podoplanin were used to stain tumor tissues and visualize blood and lymphatic vasculatures, respectively [18]. Staining was performed by a histology and imaging core facility at the University of Washington (UW). Briefly, 5 µm sections of tissue loaded onto glass slides were deparaffinized on the Leica Bond Automated Immunostainer, followed by antigen retrieval with HIER 2 (EDTA). Slides were then incubated with normal goat serum (10% in TBS) for 20 min at room temperature before incubation with a primary antibody against CD31 (rat monoclonal, Clone SZ31. Dianova, Cat. No. DIA-310, 1:250) or podoplanin (Syrian hamster polyclonal, Biolegend, Cat. No. 127402, 1:250) for 30 min at room temperature. Following primary and secondary antibody incubations, specific reactivity was detected with a Leica Bond Mixed Refine (DAB) detection kit (Leica Biosystems, Deer Park, IL, USA). Counterstaining was performed using hematoxylin. Slides were then cleared in Xylene and mounted with a synthetic resin mounting medium and #1.5 coverslips. 

ImageJ/Fiji software (version 2.9.0, Developers: Johannes Schindelin and others) was used to perform a semi-quantitative analysis of CD31 and podoplanin in axillary and sub-iliac tumors on digital images as described by Crowe and Yue [19]. Briefly, raw digital image files of IHC-stained axillary and sub-iliac tumors were imported into ImageJ/Fiji. Color deconvolution was used to isolate the positive CD31 or podoplanin stain. The extracted positive stain was then isolated in the image file and measured as pixel counts (arbitrary units).

### 2.6. Comparison of Tumor Inhibition Effects of Equivalent Doses of Gemcitabine and Paclitaxel in DcNP or Free Form

Female BALB/c mice (6–8 weeks) were inoculated subcutaneously (SC) with 1 million 4T1-luc in 50 µL HBSS on day 0. Cells were inoculated into both axillary fat pads and both sub-iliac fat pads [underneath mammary papilla (nipples) #1,4,6,9] for a total of 4 tumors per mouse. After 24 h, mice were given a single administration of saline, free drug combination (GT in DMSO/water), or GT-in-DcNP through SC injections (*n* = 4–5) in the upper abdomen area equidistant from the 4 tumors. The doses were G:20 mg/kg and T:2 mg/kg for both free and DcNP formulations (~100 µL). Early stages of cancer cell growth were monitored for bioluminescence from days 1 to 3, and untreated tumors were consistently palpable by day 5. Cancer cell growth was monitored for a total of 7 days from cell inoculation. Bioluminescent images were acquired with the IVIS as described above. Bioluminescence intensity from living mice was integrated using Live Image software.

### 2.7. Dose Dependence of Gemcitabine and Paclitaxel in DcNP on Tumor Growth Inhibition 

Female BALB/c mice (6–8 weeks) were inoculated with 1 million 4T1-luc cells SC in 50 µL HBSS on day 0. Cells were inoculated in both axillary fat pads and both sub-iliac fat pads for a total of 4 tumors per mouse. After 24 h, mice were given a single dose of GT-in-DcNP (5:0.5 mg/kg, 10:1 mg/kg or 20:2 mg/kg, G:T) or freely soluble G:T at 20:2 mg/kg. Cancer cell growth monitoring was performed with bioluminescent imaging as described above. Mouse behavior and overall health conditions were observed daily. 

### 2.8. Time Course of Gemcitabine and Paclitaxel (GT) in Tumors and Plasma in 4T1 Tumor Bearing Mouse Model and Biodistribution Study

Female BALB/c mice (6–8 weeks) were first inoculated with 1 million 4T1-luc breast cancer cells in the axillary and sub-iliac fat pads. To simplify the model, tumors were inoculated only on the left side of the mouse in the supine position (2 tumors per mouse). Sub-iliac and axillary tumors were allowed to grow until palpable or 5 to 6 days. Tumors were grown for a relatively longer period of time (5 to 6 days) to allow for the consistent excision of tumors and quantification of tumor drug concentration. Mice were then administered GT as DcNP or in free form subcutaneously under anesthesia in the center of the belly, adjacent to the sub-iliac fat pad. GT was dosed at 5:0.5 mg/kg (G:T) SC in a 20 μL (G~5 mg/mL, T~0.5 mg/mL). Blood was collected through retro-orbital bleeding at 0.083, 1, 3, 6, 24, and 48 h for DcNP and at 1 and 3 h for the freely soluble GT. In each group, three mice (based on our previous power analysis [14,15]) were used to estimate the geometric mean of the plasma concentration-time course of G and T at each time point. Necropsies were performed on each animal, and tumors were harvested at the prescribed time points for tumor drug concentration-time course studies. 

To track the time course of GT-in-DcNP particle disposition in vivo, IVIS imaging of ICG-labeled-DcNP was used according to the previous method [20,21]. ICG-labeled GT-in-DcNP (G: 5 mg/kg, T: 0.5 mg/kg, ICG: 0.075 mg/kg) was subcutaneously administered to anesthetized mice in a central area (upper belly). One hour later, the mice were euthanized, and the internal organs were exposed for fluorescence imaging by an IVIS Lumina II (Caliper Life Science, PerkinElmer; Waltham, WA, USA) (Exposure time: 3 s, F/stop: 2, Ex/Em 745 nm/820 nm).

### 2.9. Extraction of Drugs from Plasma and Tissues

Protein precipitation was used to extract G and T from plasma or tumor homogenates. Briefly, 50 µL of the sample was transferred into 1.5 mL tubes with or without dilution via a blank matrix to an appropriate concentration range. Samples were spiked with internal standards, followed by the addition of 9 volumes of acetonitrile (450 µL). Samples were then vortexed for 6 min and centrifuged at 4 °C for 15 min at 20,817 RCF. The supernatant was removed and dried under nitrogen at 40 °C. The dried samples were reconstituted to 50 µL containing 20% methanol and 80% water. 

For tumor homogenization, the density of a tumor is assumed to be equivalent to water (1 g/mL). After 5 days of growth, tumors ranged from 10–50 mg in mass. Harvested tumors were diluted in 9 volumes of PBS. The suspended tumor was then homogenized using a Dounce homogenizer due to the limited mass/volume of the sample. Tumor tissue was ground until no visible tissue aggregates could be observed. The Dounce homogenizer consisted of a borosilicate mortar (2 mL working volume) with a piston-type Teflon pestle and a stainless-steel shaft.

### 2.10. Quantification of Gemcitabine and Paclitaxel in Plasma and Tumors by HPLC-MS/MS

Gemcitabine and paclitaxel concentrations were quantified in plasma and tumor using liquid chromatography coupled with tandem mass spectroscopy (LC-MS/MS) based bioanalytical assays [14,15]. The HPLC system consisted of two Shimadzu LC-20A pumps (Shimadzu, Kyoto, Japan), a DGU-20A5 degasser, and a Shimadzu SIL-20AC HT autosampler (Shimadzu, Kyoto, Japan). The 3200 QTRAP mass spectrometer (Applied Biosystems, Grand Island, NY, USA) equipped with an electrospray ionization (ESI) TurboIonSpray source was used. The system was operated with Analyst software, version 1.5.2 (ABSciex, Framingham, MA, USA). Chromatographic separation of G and T was achieved using a Synergi column (100 × 2.0 mm; 4 μm particle size) (Phenomenex, Torrance, CA, USA) with an inline C8 guard column (4.0 × 2.0 mm) also from Phenomenex. The flow rate was set to 0.5 mL/min with a 5 µL sample injection volume. The mobile phase for separation consisted of pump A (20 mM Ammonium Acetate in water) and B (Reagent Alcohol). The gradient program used was as follows: pump B was maintained at 20% for 1.0 min, then increased to 97% at 2.0 min, held at 97% until 3.0 min, ramped to 3% by 4.0 min, and held until 5.5 min. The needle was washed with isopropanol after each injection. Analytes were monitored using multiple-reaction monitoring (MRM) for positive ions. The following ion transitions were monitored: gemcitabine, *m*/*z* 264.066→112.000; paclitaxel, *m*/*z* 854.266→286.200; a stable labeled isotope of gemcitabine (C_8_^13^CH_12_ClF_2_^15^NN_2_O_4_) (*m*/*z* 267.067→115.100) was used as an internal standard for gemcitabine. Docetaxel (*m*/*z* 830.312→549.3) was used as an internal standard for paclitaxel.

### 2.11. Effect of GT-in-DcNP on Human MDA-231-HM Tumor Implanted in Fat-Pad of Athymic Mice

To verify whether GT-in-DcNP effects can extend to human breast cancer, we have evaluated GT-in-DcNP effects in the MDA-231-HM tumor model. MDA-231-HM (a luciferase transfected brain metastasis derivative of MDA-MB-231 from the City of Hope National Medical Center) [17] was obtained and implanted into the mammary fad pads of athymic nude mice (NU/J or Foxn1nu, The Jackson Laboratory). the MDA-231-HM cells were inoculated in 4 mammary glands (#1,4,6,9) of athymic mice under anesthesia. Like the murine 4T1 breast cancer cells, human MDA-231-HM cells stably express the luciferase enzyme, which can convert luciferin to luminescence, and thus, bioluminescence intensity (BLI) can be used to monitor tumor growth. A total of 10 nude mice were inoculated with 2 million cells in each of the 4 mammary fat pads near the axillary and sub-iliac lymph nodes. These 10 mice bearing cancer nodules were treated with either saline (negative control, n = 4, 16 total tumors) or GT-in-DcNP (test article, n = 3, 12 total tumors). Having 4 tumors in each mouse provided sufficient power to discern the effects of GT-in-DcNP compared to the placebo control group. Mice were given a single, high dose on day 1 at 20:2 (G:T) mg/kg, followed by maintenance doses of 10:1 (G:T) mg/kg on days 7, 14 and 21. Tumor growths were monitored by IVIS bioluminescence, as described above. In the preliminary experiment and placebo-treated mice, we found that in MDA-231-HM inoculated mice, the bioluminescence value declined for the first 3 days before tumor development and subsequently grew in the aforementioned mammary glands. Thus, the treatment effects on bioluminescence data were presented accordingly. 

### 2.12. Statistical Analysis

All values were presented as mean ± standard deviation wherever applicable. The number of mice in all groups ranged from 3 to 10. Students’ *t*-tests were performed to determine statistical significance between two groups, and two-way ANOVA was used to evaluate differences between treatment groups over time. A *p*-value of <0.05 was considered statistically significant. Statistical analyses were performed using GraphPad Prism (Version 7.0).

## 3. Results

### 3.1. Characterization of a Primary Multi-Site Mammary Fat Pad 4T1 Breast Cancer Model 

To evaluate the treatment effects of targeted and synchronized GT combination therapy in the early stages of metastatic breast cancer, we have investigated a number of metastatic breast cancer models that can consistently form tumors in breast tissue. We found that inoculation of the aggressive metastatic 4T1 breast cancer cell line consistently produces breast tumors in the mammary fat pads of BALB/c mice. Therefore, we used this orthotopic, syngeneic 4T1 breast tumor model to investigate the effect of GT-in-DcNP. We first evaluated whether 4T1 breast tumors in multiple mammary fat pads can provide multiple distinct tumors in each mouse for assessment of therapeutic effect, although there were regional differences in tumor growth. 

To accomplish the goal, 4T1-luc cancer cells were first inoculated in four mammary fat pads of each mouse: two adjacent to the proper axillary lymph nodes and two adjacent to the sub-iliac lymph nodes. The axillary and sub-iliac fat pads were inoculated symmetrically across the midline of the mouse in the ventral medial view (Figure 1A). We found that in the first 3 days, there was no palpable or notable tumor mass in any site of inoculation. During this period, the tumor establishment and growth were detected as luminescence of 4T1-luc based on bioluminescence imaging of the 4T1 transfected with a luciferase marker. 

In a pilot study, we determined the effects of 4T1 cell number (0.2, 0.5, or 1 million in 20 µL, 50 µL, or 100 µL, respectively) on tumor establishment and growth in the mammary tissues. In a single mouse bearing four tumors, we found that higher doses of 4T1 cells (0.5 and 1 million) could produce a bioluminescent signal as early as one hour after injection (day 0). The bioluminescent signals were dose-proportional across four tumors in single mice with 1 million (2065 ± 877, mean ± S.D of 4 tumors) and 0.5 million cell inoculations (1025 ± 940, mean ± S.D) one hour after injection. No signal above baseline (>500 counts) was detected in the mouse administered 0.2 million cells until 24 h after injection (760 ± 894, mean ± S.D), with stable bioluminescence observed in the higher dose levels (Table 1). Thus, 0.5 million 4T1 was identified as a minimal inoculant, but 1 million 4T1 cells are needed to ensure consistent tumor take in mammary fat-pads in mice.

We next determined whether an inoculant of 1 million 4T1 cells in 50 µL (instead of 100 µL) volume would provide similar results on a breast tumor profile in the fat pad and reduce the tissue back pressure from injection. The data summarized in Table 1 indicate that while there are some variations between the two experiments on day 1, by day 3, an approximate 5–6-fold consistent increase in luminescence was observed, and the tumor volume appeared to be in the range of 40–53 mm^3^ for both volumes of cell inoculant. The follow-up studies thus demonstrated the reproducibility and robustness of the model. In these studies, 4T1 inoculated mice were monitored for up to 7 days to aid in the design for the subsequent treatment intervention studies. The tumor progression time-course is presented in Figure 1B. As shown in Figure 1B, distinct tumor growth rates in fat-pads at axillary and sub-iliac sites were notable. The tumor growth rates, based on tumor cell luminescence in the sub-iliac location, were significantly lower than those in the axillary sites. By day 7, 2.3-fold higher tumor luminescence was notable in axillary tumors as compared to that of sub-iliac tumors. The overall tumor volume analysis was also consistent and reproducible for each region (axillary and sub-iliac) in the replicated studies, despite biological variation in 4T1-dependent luminescence on day 1 (Table 1, Figure S1). Tumor growth was consistent across the midline of the mouse (left and right sides), but significant differences were noted between the axillary and sub-iliac fat pads (Figure 1B), *p* < 0.05 2-way ANOVA). These differences were responsible for the inter-tumor variability observed in Table 1. 

To further characterize the differences in tumor growth from axillary and sub-iliac inoculations, tumors were resected at a fixed time point (5 days) for physical measurement. After 5 days of growth, the weight of 20 tumors was compared using a Student’s *t*-test (mean ± S.D). For axillary tumors, no difference was observed in tumor mass across the midline (left axillary, 76.9 mg ± 25.8 mg; right axillary, 86 mg ± 41.1, *p* = 0.7). For sub-iliac tumors, no difference was observed across the midline (left sub-iliac, 46.9 mg ± 10.8 mg; right sub-iliac, 36.4 mg ± 18.5, *p* = 0.3). However, in comparing the axillary versus sub-iliac 4T1 tumor masses, we found that the axillary tumors were significantly larger than the sub-iliac (left axillary vs left sub-iliac, *p* = 0.04; right axillary vs right sub-iliac, *p* = 0.04); these data are consistent with the tumor luminescent measurements. Based on these results, mice inoculated with 1 million 4T1-luc cells in 50 µL volume are used for the treatment intervention experiments with GT drug combination in DcNP or soluble dosage form.

### 3.2. Characterization of Lymph and Blood Vasculature Development in Primary 4T1 Tumors

To determine the role of lymphatic systems in supporting 4T1 tumor growth in the mammary fat pads, we evaluated tumor vasculature harvested from a mouse with mammary fat-pad tumors on day 5. While it would be of interest to investigate cancer vasculature at earlier time points (days 1 to 3), the lack of a palpable tumor limits the ability to harvest those tissues at early time-points. Tumor tissues were harvested and fixed in 10% formalin buffered saline and visualized with immunohistochemistry to evaluate lymph and blood tumor vasculatures. Antibodies specific to podoplanin markers for lymphatic vasculature and CD31 for blood endothelial cells were used for tumor vasculature characterization [18].

**Figure 2:**
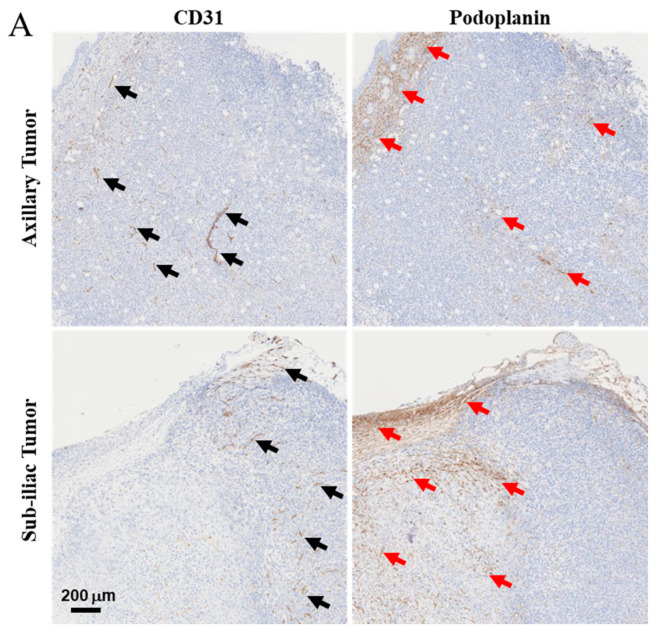

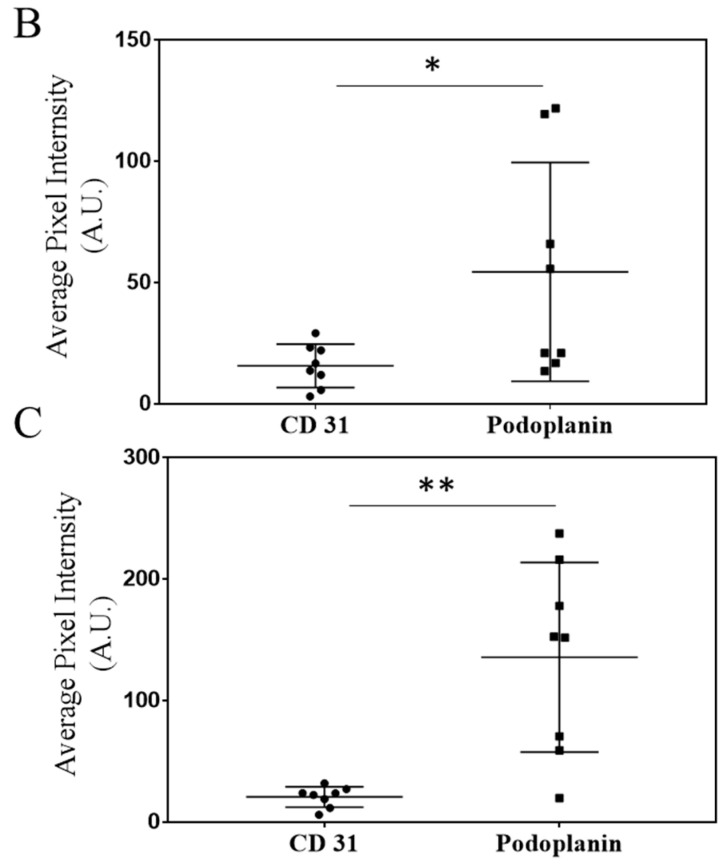
Characterization of blood and lymphatic vasculature development in growing primary tumors. After 5 days of growth, 4T1 tumors harvested from the axillary and sub-iliac fat pads displayed vascularization with both blood and lymphatic vessels. CD31 was used as a marker for endothelial cells on blood vessels and is highlighted by black arrows. Podoplanin was used as a marker for the lymphatic vascular system and is highlighted by red arrows. Representative IHC slides from the tumor harvested from fad-pads of mice from axillary and sub-iliac sites are presented in Panel (**A**). ImageJ analysis was performed as a semi-quantitative measure of positive staining for CD31 (black circle) and podoplanin (black square) in the axillary (Panel (**B**)) and sub-iliac tumor (Panel (**C**)). * *p* < 0.05, ** *p* < 0.001, Student’s *t*-test. Data presented in Panels (**B**,**C**) are based on the mean ± SD of eight randomly selected 4T1 tumor areas from each site.

Histological analysis of axillary and sub-iliac tumors reveals that on day 5, both tumors exhibited well-vascularized lymphatic (podoplanin marker) and blood (CD31 marker) vessels. Using the ImageJ software, histological slides were analyzed to determine the relative intensity of staining by CD31 or podoplanin for a semi-quantitative comparison of lymphatic and blood endothelial vasculature. Axillary tumors had a higher degree of staining for podoplanin compared to CD31 (54 ± 15 AU versus 16 ± 3 AU, *p* < 0.05), suggesting a higher degree of lymphatic vessels surrounding 4T1 tumors. Similarly, sub-iliac tumors also had a higher degree of staining for podoplanin versus CD31 (136 ± 27 versus 21 ± 3, *p* < 0.05). The data presented in Figure 2B and Figure 2C respectively, suggest that higher frequencies of lymphatic vasculature compared to that of blood are noted in tumor-laden fat-pads on day 5.

### 3.3. Effects of DcNP on Gemcitabine and Paclitaxel Combination to Inhibit 4T1 Mammary Tumor

To evaluate the effects of DcNP on gemcitabine (G) and paclitaxel (T) as a fixed-dose combination (10:1 *w*/*w*, approximate to the clinical regimen), we first prepared GT-in-DcNP as previously described [14,15]. The GT-in-DcNP particles were previously identified as non-liposomal, lozenge shape particles without apparent membrane structure. The average hydrodynamic diameter was ~60 nm, measured by a DLS particle analyzer. The average zeta potential of DcNPs was measured to be −16.4 mV at pH 7. The drug association efficiencies (AE%) to DcNP in suspension under sink conditions (dialysis) were determined to be ~10% for G and ~100% for T [15]. The GT-in-DcNP did not exhibit a burst release of either drug over the 4 h dialysis period. Additionally, the GT-in-DcNP nanoparticle suspensions were stable at room temperature for at least 3 months without significant changes in these physicochemical properties or a change in AE%. Through mechanism-based pharmacokinetic modeling, the in vivo drug associations to DcNP through IV injection were determined to be ~98% and ~75% for G and T, respectively [14,15]. The in vivo drug release rates (at 37 °C in mice) were estimated to be 0.2 h^−^^1^ for G and 1.9 h^−^^1^ for T through the modeling approach [14,15].

**Figure 3:**
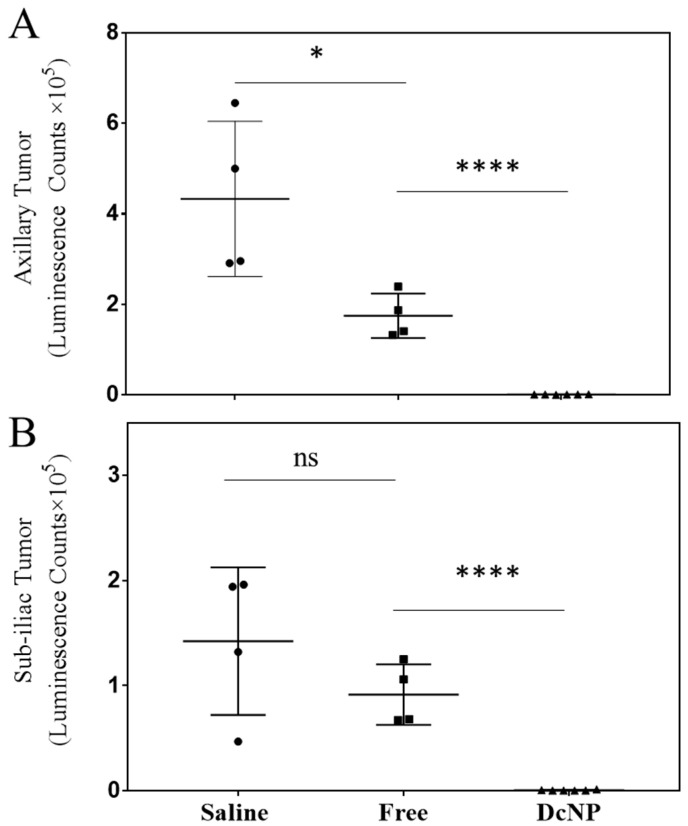
Effect of DcNP on tumor inhibition by gemcitabine and paclitaxel when compared to the same dose of free drugs. Mice were treated with either saline (black circle), GT in DMSO (20/2 mg/kg, black square), or GT in DcNP (20/2 mg/kg, black triangle) 24 h after cancer cell inoculation. Mice were monitored for 7 days, and tumor growth was evaluated by luminescence. The integrated luminescence photon counts in the axillary and sub-iliac tumors are presented in panels (**A**) and (**B**), respectively. The mean and standard deviations of luminescence (*n* = 4–6) are presented above. ns: non-significance, * *p* < 0.05, **** *p* < 0.0001, Student’s *t*-test.

We next determined the treatment effects of the breast cancer drug combination, gemcitabine and paclitaxel (typically used in metastatic breast cancer treatment), on 4T1 tumors. As shown in Figure 3, by day 7, mice treated with free GT control exhibited significant effects on 4T1 tumor growth with a 60% reduction compared to placebo (saline: 309,154 ± 184,740 vs. Free GT: 123,994 ± 63,419 counts, *p* < 0.05) in the axillary tumor and 52% reduction in the sub-iliac tumor (Saline: 132,450 ± 79,333 versus Free GT: 64,743 ± 36,131 counts, *p* < 0.05). Compared to the free dosage form, GT-in-DcNP surprisingly produced a much greater inhibitory effect on tumor growth with an additional 99.7% reduction (878.8 ± 450.8, *p* < 0.001 compared to the free dosage form) for the axillary tumor and an additional 99.5% reduction (608.3 ± 436.8, *p* < 0.0001 compared to free dosage form) for the sub-iliac tumor. Collectively, these data suggest that the DcNP significantly enhanced GT’s ability to inhibit 4T1 tumor growth in the fat pad after a single subcutaneous G:T 20:2 mg/kg dose compared to the free drug combination dosage form. 

### 3.4. Dose–Response Study of Gemcitabine and Paclitaxel in DcNP Form to Suppress 4T1 Breast Tumor

**Figure 4:**
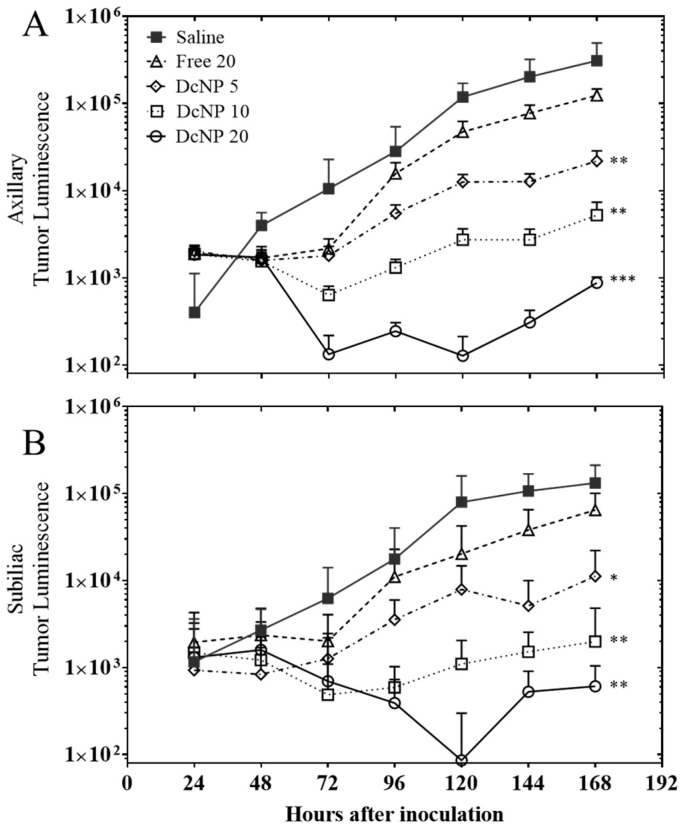
Dose proportional effects of gemcitabine and paclitaxel in DcNP or free form on orthotopic 4T1 tumor inhibition. Mice with 4T1 tumors in fat-pads were treated with GT in free form at 20:2 mg/kg (∆) or GT-in-DcNP at 5:0.5 mg/kg (◊), 10:1 mg/kg (□) or 20:2 mg/kg (○) 24 h after cancer cell inoculation. Mice were monitored for 168 h (7 days) after cell inoculation, and before termination, and tumor growth was evaluated by luminescence. Panel (**A**) and (**B**) represent the luminescence signal integrations from axillary tumors and sub-iliac tumors, respectively. The mean and standard deviations of luminescence are presented (*n* = 8–10). DcNP vs. Free, * *p* < 0.005, ** *p* < 0.01, *** *p* < 0.005, Student’s *t*-test.

To elucidate the relationship between increasing dosage on efficacy of GT-in-DcNP, we next performed a dose-ranging study with GT-in-DcNP. Mice inoculated subcutaneously with 1 × 10^6^ 4T1 cells were given gemcitabine and paclitaxel in DcNP at G:T 20:2; 10:1; or 5:0.5 mg/kg (referred to as DcNP 20, 10, 5) as a single subcutaneous dose and monitored for 7 days. Tumor growth for each mouse was measured and is presented in Figure 4. A 20:2 mg/kg dose of GT was chosen for both free and DcNP dosage forms. This dose level was selected because it resulted in nearly complete suppression of tumor growth when administered as GT-in-DcNP in the previous study (Figure 3). The time course data from a single-dose study has been incorporated into the dose–response study to improve the statistical power.

As shown in Figure 4, 4T1 inoculated mice produced a 1000-to-10,000-fold increase in tumor cell luminescence over the 7-day study. At the lowest GT-in-DcNP (5:0.5 mg/kg) dose, tumor growth was inhibited from day 1 to day 3. On day 4, mice administered with 5:0.5 mg/kg GT-in- DcNP had a tumor signal ~3.5-fold the baseline value (day 1), reflecting limited cancer growth and rebound. The dose–response effect on tumor regression is notable at 10:1 mg/kg, with GT-in-DcNP treated mice exhibiting tumor luminescence below that of day 1. When the dose–response effects on the axillary and sub-iliac tumor growth patterns (over time course) were normalized to the baseline luminescence on day 1, the tumor suppressive effects were notable for days 3–5 for both tumors implanted in axillary and sub-iliac sites (Figure 4). In contrast, in mice treated with the highest (20:2 mg/kg) GT in free-soluble dosage form, there was a brief reduction in tumor growth compared to saline-treated mice, but a rapid rebound was observed with a subsequent growth profile similar to that of saline. Collectively, these data suggest that GT formulated in DcNP provides a significant tumor burden reduction in a dose- and time-dependent manner at levels that were not achievable with free-soluble GT given in fixed-ratio (10:1 *w*/*w*).

### 3.5. Mechanisms Relating to DcNP Mediated Enhancement in GT Exposure Leading to 4T1 Mammary Tumor Regression and Inhibition

To determine whether subcutaneously administered GT-in-DcNP can preferentially be taken up into the lymph vessel and subsequently distributed and retained in multiple lymph nodes, we first labeled GT-in-DcNP with an infrared marker, indocyanine green (ICG) [20]. We then followed GT-in-DcNP ICG infrared fluorescence over time after subcutaneous dosing as described previously [21]. These mice with 4T1 breast tumors were also evaluated for enhanced drug co-localization to evaluate whether synchronized delivery may play a role in DcNP-mediated enhanced anti-tumor activities. The following summarizes the results of these studies.

#### 3.5.1. Effects of Drug Combination Nanoparticles on Gemcitabine and Paclitaxel Transit and Accumulation in Lymphatic Vessels and Tumors

**Figure 5:**
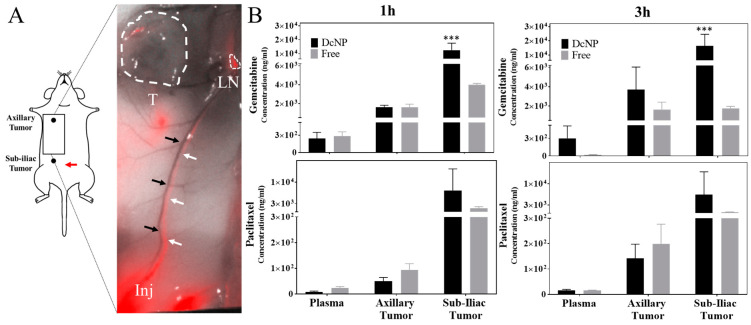
Effect of DcNP on the transit and accumulation of gemcitabine and paclitaxel in lymphatic vessels and tumors following subcutaneous injection. Panel (**A**): ICG infrared fluorescence-labeled GT-in-DcNP were subcutaneously injected at the lower abdomen area of tumor-bearing mice (red arrow). At 1 h, the ICG-fluorescence signal from GT-in-DcNP is detectable at the injection site (Inj) and throughout the lymphatic vessels (white arrows), but not in the blood vessel (no detectable fluorescence)—in the thoracoepigastric vein (black arrows), which is visible as a dark vessel located parallel to the fluorescent lymphatic vessels. The fluorescence signal can also be observed in the axillary node (LN) with small traces on the surface of the tumor (T). Panel (**B**): The corresponding GT drug concentrations in the tumors and plasma are presented for the 1-h and 3-h time points for mice dosed with DcNP or soluble GT at an equivalent dose. The data presented were mean + SD. *** indicates *p* < 0.005.

To investigate whether subcutaneously administered GT-in-DcNP can preferentially be distributed, first into the lymph vessels and then into the lymph nodes, we prepared GT-in-DcNP labeled with indocyanine green (ICG) as a tracer for tracking GT-in-DcNP particles in vivo. The final ICG-labeled GT-in-DcNP characteristics were similar to that of GT-in-DcNP without a label. Having ICG serving as an infrared marker for GT-in-DcNP, biodistribution time-course can be performed based on infrared imaging analysis. In previous work, hydrophobic ICG was incorporated, stably, as an infrared marker into DcNP nanoparticles (containing HIV drugs) to track DcNP in mice after subcutaneous injection, and results indicate that DcNP drains rapidly from the subcutaneous injection site and is distributed first to local lymph nodes and then subsequently to nodes throughout the body [21] as fluid in the subcutaneous space drains to lymphatic vessels and nodes. In this study with GT-in-DcNP, we administered subcutaneously GT-in-DcNP containing ICG tracer at a central-abdominal injection site (the belly), in tumor-bearing mice. The subcutaneous abdominal injection site was chosen to capture the upward flow of lymphatic fluid from the sub-iliac lymph node to the axillary lymph node and throughout the lymphatic system, as previously reported [22,23]. As shown in Figure 5, one hour after a subcutaneous injection of ICG labeled GT-in-DcNP (G: 5 mg/kg, T: 0.5 mg/kg, ICG: 0.075 mg/kg), an ICG fluorescent signal can clearly be observed transiting from the injection site (adjacent to the sub-iliac lymph node) to the axillary region of the mouse. The ICG fluorescent signal runs parallel to the thoracoepigastric vein (Figure 5A, black arrows) and shows the transit of ICG-tagged GT-in-DcNP through the lymph vessels (Figure 5A, white arrows) and not within the adjacent blood vessel (black arrows). Importantly, ICG-labeled GT-in-DcNP can also be detected on the surface of the axillary tumor (Figure 5A, T), suggesting that GT-in-DcNP have direct access to tumors through the lymphatic vessels throughout the body as these particles distribute to the nodes within the axillary and iliac regions.

Next, a quantitative analysis of gemcitabine (G) and paclitaxel (T) concentrations in tumors and plasma was performed. While infrared fluorescence tracing of ICG-labeled DcNP allowed us to track the route of distribution, a quantitative analysis of GT concentrations in the tumor allows us to confirm that the drugs are also transiting to the site of action. Therefore, we next measured GT drug concentrations in tumors and plasma. For this purpose, we have chosen two early time points, 1 and 3 h, to allow the detection of drugs in the mice receiving control (soluble) and test (DcNP) formulations. In mice treated with free gemcitabine, a decrease in plasma and sub-iliac tumor concentrations was observed over time, while no change was seen in the axillary tumor (Figure 5B). In contrast, mice treated with GT-in-DcNP exhibited increasing gemcitabine concentrations from 1–3 h for both axillary and sub-iliac tumors. There is also a small (but not significant) increase in plasma G concentration in mice treated with GT-in-DcNP. Interestingly, the behavior of paclitaxel administered as DcNP did not differ significantly from the free drug.

#### 3.5.2. Ability of DcNP to Enhance Gemcitabine and Paclitaxel Retention in Tumors and Extend Persistence of Plasma Drug Concentrations

**Figure 6:**
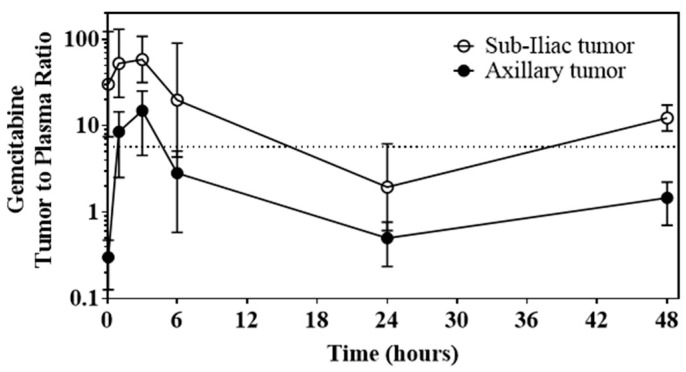
Time-course of GT-in-DcNP to sustain tumor gemcitabine levels as determined by tumor-to-plasma gemcitabine ratio. GT-in-DcNP administered subcutaneously resulted in consistent tumor-to-plasma concentration ratios of gemcitabine near or above 1 for 48-h in both sub-iliac and axillary 4T1 tumors implanted in fat-pads.

To determine the extent of enhanced tumor distribution from the DcNP formulation, GT drug concentrations in blood and tumors from mice treated with GT-in-DcNP were measured and analyzed as tumor-to-blood ratios at 0.083, 1, 3, 6, 24, and 48 h. These data are summarized in Figure 6. Tumor-to-plasma gemcitabine concentration ratios were consistently above or around 1 when GT-in-DcNP was administered subcutaneously for 48 h in both sub-iliac and axillary 4T1 tumors implanted in fat-pads, meaning over the course of the experiment, the drug concentrations were maintained higher in the tumors than in the blood.

### 3.6. Safety and Pathology of DcNP Effects on GT Drug Combination Treatment

To evaluate safety, we also monitored animals daily for signs and symptoms of toxicity from the dose–response study. Local toxicity was dose-proportional and manifested as injection site erythema, which did not affect mouse ambulation, behavior, or activity. No edema was observed but injection site erythema was present and increased in severity with dose [24]. Only one of five mice given 20:2 mg/kg GT-in-DcNP showed some sign of lethargy and reduced body weight on day 7, but no erythema or necrosis at the injection site was observed. At necropsy, the gastrointestinal (GI) tract of one mouse was slightly bloated and inflamed, which may suggest damage to the epithelium, commonly observed with antimetabolite therapy [25]. In the other four mice that received 20:2 mg/kg GT-in-DcNP, no weight loss or change in behavior was observed despite the dermal reactions (Table S1).

**Figure 7:**
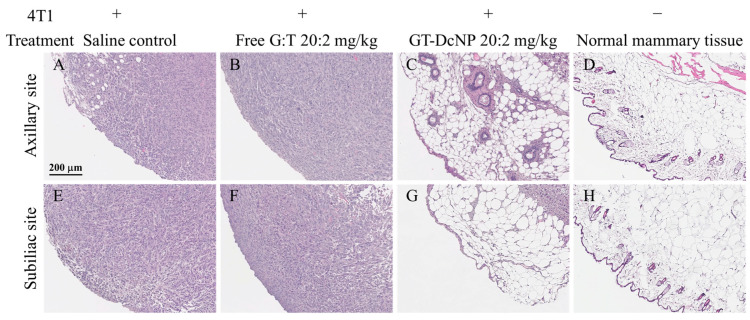
Marked reduction of neoplastic 4T1 cells in tumor sites, with retention of normal mammary tissue elements following treatment with GT-in-DcNP and not free GT. The remaining tumor masses from mice inoculated with 4T1 cells were surgically resected 6 days after treatment with saline (**A**,**E**), free drugs (20:2 mg/kg) (**B**,**F**), or GT-in-DcNP (20:2 mg/kg) (**C**,**G**). The histology of healthy mammary fat-pads from non-cancer mice is presented in panel (**D**,**H**). The resected axillary site tumors or normal mammary tissue are presented in the top (**A**–**D**) panel, and sub-iliac site tumors or normal mammary tissue are presented in the bottom (**E**–**H**) panel. Tissues were fixed in 10% neutral-buffered formalin.

On day 7, the same end point as the dose-ranging study, the histopathologic findings from the tumor-bearing fat-pads of mice treated with GT in free and DcNP dosage forms were compared and are presented in Figure 7. Representative tumors and surrounding tissue from the free drug combination (20:2 GT mg/kg) and saline-treated mice (placebo control) show large, dense foci of 4T1 cells present in most areas examined (Figure 7A,B,E,F), and are consistent with bioluminescence measurements. In contrast, representative tumor implanted fat-pads isolated from mice treated with an equivalent dose of GT-in-DcNP exhibited tissue characteristics with only small, dispersed foci of 4T1 tumor cells (Figure 7C,G). These tissues appeared to have preserved most elements of normal mammary glands, including adipose and ductular tissues (Figure 7D,H). These histological findings are consistent with the tumor growth profile, and treatment effects on the reversal of fat-pad tissues are notable in Figure 3.

## 4. Discussion

Early detection and intervention in breast cancer, coupled with advances in breast cancer therapies, have enabled many patients to achieve remission with improved life-quality. However, even with the best available targeted treatment regimens, many patients eventually progress to metastatic breast cancer. Metastatic progression is partially due to drug resistance and heterogeneous cancer outgrowth. At the metastatic stage, highly potent and broad-acting chemotherapeutic agents are typically prescribed but may require multiple drugs given in combination to extend life. While combination chemotherapies bind to various targets in tumor cells to maximally inhibit cancer growth and replication, current dosing regimens lead to asynchronous tissue and cell exposure and non-specific distribution to healthy by-stander tissues and cells. As a result, even with highly potent chemotherapeutics such as gemcitabine and paclitaxel, the therapeutic potential is limited by off-target toxicities to healthy organs, which leads to intolerable and untoward effects in patients.

With the successful assembly of two physically incompatible drugs, gemcitabine (water soluble) and paclitaxel (water insoluble), into stable drug-combination nanoparticles that co-localize in breast cancer tumors, we have tested whether the combined delivery of GT can improve the effectiveness of the treatment regimen. We further characterized the dose–response and potential mechanisms leading to the enhanced therapeutic outcomes of GT-in-DcNP in the orthotopic 4T1 breast tumor model. Our results demonstrate that DcNP provides stability in vivo, resulting in a synchronized gemcitabine and paclitaxel localization in tumor tissues (Figure 5A) and cells (Figure 5B) at levels that cannot be achieved by free-drug combination. In addition, a single subcutaneous dose of GT-in-DcNP leads to enhanced and synchronized drug levels for both gemcitabine and paclitaxel in the tumors from the four mammary fad pads at axillary and sub-iliac locations. Time-course tracking of DcNP based on infrared marker ICG labeled GT-in-DcNP also supported this hypothesis as GT-in-DcNP are taken up into the lymph after subcutaneous dosing (Figure 5). Once in the lymph, the DcNP appear to be stable as they traverse over time through lymph vessels throughout the body; thus, it is likely that GT-in-DcNP after subcutaneous dosing is taken up predominantly into lymph vessels and distributed subsequently through the lymphatic system in this 4T1 orthotopic tumor model.

It is remarkable that a single dose of GT-in-DcNP, but not the equivalent dose of free GT, is able to suppress greater than 99.5% of 4T1 tumor growth. In comparison, the highest dose of free GT (20:2 mg/kg dose) exhibited 51.1–59.9% tumor suppression on day 7 (Figure 3). Even at a much lower 5:0.5 mg/kg dose, GT-in-DcNP exhibited about 6-fold lower tumor size compared to those treated with the higher 20:2 mg/kg dose of free GT combination. Additionally, the time-course tumor luminescence tracking demonstrates that single-dose SC GT-in-DcNP can achieve tumor regression from days 3 to 5 before a slight rebound, which may be due to insufficient long-acting drug coverage at sustained therapeutic levels (Figure 4).

In developing a multi-site tumor model, it was first noted that tumors inoculated in different mammary fat pads had different growth patterns that may be related to regional differences between axillary and sub-iliac breast tumors established in mice (Figure 1). Tumor size, weight, and bioluminescence measurements were all consistent and in agreement with the overall trend of tumor growth. Based on these results, there are likely biological factors, including blood and nutrient supply, as well as other factors that may support tumorigenic activities that lead to the notable differences in the 4T1 tumor profiles between the axillary and sub-iliac sites. This observation is in line with other reports that described the dependence of the inoculation sites on tumor growth rates [26]. Nevertheless, these results indicate that a 4T1 breast cancer model implanted in multiple mammary fat pads at axillary and sub-iliac sites can lead to consistent tumor take and predictable growth profiles amenable for evaluating therapeutic effects. By implanting at two sites, each in axillary and sub-iliac fat pads of the mouse, one can increase the tumor number (and perhaps experimental power) to discern treatment effects with minimum animal numbers. With cancer bioluminescent tracking provided by 4T1-luc cells, the overall time-course of breast tumor growth can be monitored in this orthotopic tumor model. In addition, the stratification of tumors into rapid-growing (axillary) and slow-growing tumors (sub-iliac) may be useful for the therapeutic evaluation of GT by providing measures of effect on both fast and slow-growing tumor types.

Histological analysis of axillary and sub-iliac tumors suggests that blood and lymph vasculature surrounding the 4T1 tumors were developed within 5 days (Figure 2). The significantly (>4–6 fold) higher lymphatic than blood vessels when verified with respective lymph vs blood endothelial cell-markers is notable. The higher tumor-associated lymphatic vasculature, compared to blood vessels, is found in the 4T1 tumor fat pads for both axillary and sub-iliac sites. The lymphatic vasculature becomes an opportunity for cytotoxic drugs in DcNP formulation to preferentially deliver and accumulate in tumors. Then, GT-in-DcNP treatment may be effective not only in suppressing tumor growth at the primary site but also in lymphatic-mediated metastases. While it remains to be directly tested, it is possible that abundant tumor-associated lymphatic vessels (Figure 2) may enhance GT-in-DcNP uptake and accumulation in the breast tumor through the lymphatic vessel that supports rapid tumor growth. The enhanced and preferential lymphatic uptake, localization in nodes, and distribution to tumors throughout the body of GT-in-DcNP may be related to the 99.5% to 99.7% tumor reduction compared to the equivalent dose of GT-free drug combination (51.1% to 59.9% tumor reduction) (Figure 3). Furthermore, a lower dose (5:0.5 mg/kg) of GT-in-DcNP showed greater inhibition of tumor growth compared to a four-fold higher dose of free combination GT, supporting the hypothesis that longer retention extends the pharmacological activity of GT-in-DcNP much greater than can be achieved with free drug. With these promising results, we further investigated the potential mechanism of tumor targeting achieved with GT-in-DcNP by first studying the drug distribution and accumulation throughout the lymphatic tissues after subcutaneous administration.

The concentration-time profiles of GT given in free versus DcNP form corroborate our qualitative observations in Figure 5. As DcNP follows the upward flow of lymphatic fluid toward the thoracic duct, gemcitabine comes into contact with the sub-iliac and axillary tumors. On the other hand, the stability of GT-in-DcNP also provides extended circulation time to reach tumors near the axillary nodes (a lower part of the body where DcNP is distributed against the natural lymphatic flow direction). Due to the lack of clearance mechanisms for gemcitabine in the lymph, this slow transit is reflected by the steadily increasing concentrations of gemcitabine in the sub-iliac and axillary tumors from 1 to 3 h. Gemcitabine, bound to particles, is then slowly released into the blood (as reflected by the increasing plasma concentrations) through the thoracic duct. In the free gemcitabine treatment, the drug can be taken into the systemic circulation or tumor tissues but is instantly available for metabolism (as reflected by decreasing plasma concentrations). Although there is a slower decline of free gemcitabine in tumors compared to plasma, this slower decline could reflect tissue retention. Nevertheless, the tumor concentrations of gemcitabine at 1 and 3 h are consistently higher when given in DcNP form compared to free form. Over 1 and 3 h, formulating GT-in-DcNP had a significant effect on the time course of gemcitabine when compared to the free drug. It is interesting to note that gemcitabine levels in tumor tissues from the axillary site were consistently lower than that found in the sub-iliac site throughout. In addition, the tumor-to-plasma ratios were greater than 1 for all time-points, indicating that higher tumor gemcitabine accumulation appeared to be mediated by the DcNP dosage form. Due to the 10-fold lower dose of paclitaxel compared to gemcitabine, paclitaxel levels in plasma and tumors fell below the detection limits of our drug assay within 6 h. Persistent paclitaxel concentrations in mice dosed with GT-in-DcNP were detectable in the sub-iliac tumors for up to 48 h. A pooled sample or higher dose pharmacokinetic studies, which is beyond the scope of this report, may be needed to further characterize paclitaxel blood, tissue, and tumor kinetics. Collectively, these data show that GT-in-DcNP drug combination particles were taken-up and distributed through the lymphatic vessels to tumors in the fat pads, resulting in higher concentrations of gemcitabine in axillary and sub-iliac tumors compared to that of free GT dosage form. The fixed dose and synchronized accumulation and exposure in cancer tissues and cells may be related to the high degree of tumor growth suppression leading to apparent but transient tumor regression of 4T1 tumor in the mammary fat pads.

While the exact mechanism of tumor targeting achieved by GT-in-DcNP is not clear and remains to be elucidated, it is likely that it is due to novel characteristics of the nanoparticle. GT-in-DcNP are able to selectively penetrate the porous lymphatic vessels. Upon entering the lymph vessels, one can envision that GT-in-DcNP will be trapped and distributed throughout the lymphatic systems, including the nodes and other lymph-accessible tissues. Due to their size and shape, DcNP particles have been shown to localize within the sinuses of the nodes, and their retention provides long-acting drug-kinetics in vivo [27,28]. With the reported stability of GT-association to DcNP particles, it is possible that the sinuses of the lymph nodes surrounding the tumor tissues may provide a high degree of drug exposure to the tumor. We envisioned that the tumor-laden breast tissues may also increase the sinuses of enlarged lymph nodes in response to tumor growth [29,30]. A combination of these mechanisms may lead to the enhancement of DcNP to provide higher GT concentrations and longer retention near or within the tumor for mediating tumor mass reduction. The finding of higher lymphatic presence in the 4T1 breast tumor tissues (Figure 2) supports this mechanism, which leads to enhanced GT anti-tumor effects provided by DcNP.

The ability of DcNP to enhance the effect of GT on tumor growth and tumor-size reduction through a lymphatic trafficking mechanism is an exciting discovery, but limitations still exist in the current study. The syngeneic model in this study only uses 4T1 murine breast cancer tumors to study the efficacy of 10 to 1 GT-in-DcNP subcutaneous treatment, but human breast cancer cells may have different sensitivity to these two drugs compared to mouse breast cancer tumor cells. Some cells may be more sensitive to paclitaxel than 4T1 [31]. As a result, a lower G-to-T ratio may be needed. Thus, additional studies are needed to define the optimal GT fixed-dose combination that may be safe and effective for a range of breast cancers with different drug sensitivities; however, exploration and optimization of GT ratio in DcNP formulation, while important, is beyond the scope of this report and is a subject of our active investigation.

**Figure 8:**
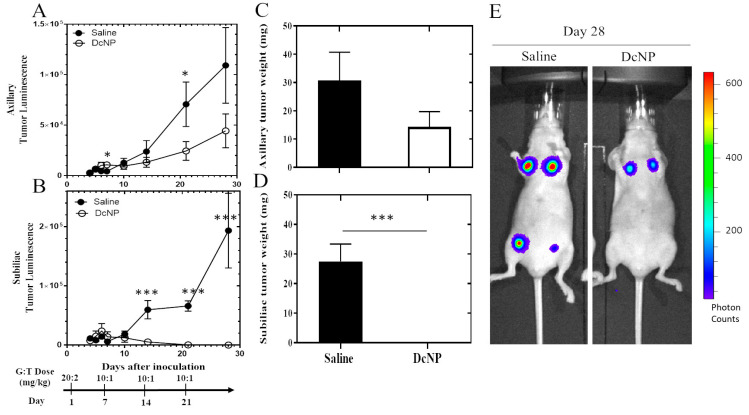
Effect on GT-in-DcNP treatment on human breast cancer MDA-231-HM orthotopic mouse model. Athymic mouse inoculated with 2 million luciferase-transfected MDA-231-HM human breast cancer cells. These mice were then treated with a placebo (control) or GT-in-DcNP (test treatment product) by subcutaneous injection on days 1, 7, 14, and 21. The G:T dose given was 20:2 mg/kg on day 1 and 10:1 mg/kg on days 7, 14, and 21. The effects of GT-in-DcNP or placebo treatment were presented as two-panels. These data present the bioluminescence intensity starting on day 4 to monitor MDA-231-HM tumor growth at the axillary (Panel (**A**)) and the sub-iliac (Panel (**B**)) locations. DcNP treatment effects on tumor weights were compared to placebo treatments for MDA-231-HM tumors isolated from the axillary (Panel (**C**)) and sub-iliac (Panel (**D**)) sites on day 28. * *p* < 0.05, *** *p* < 0.001 based on Student’s *t*-test. Panel (**E**) presents bioluminescence images of MDA-231-HM tumors from saline control and GT-in-DcNP groups on day 28.

We have also conducted a preliminary study to evaluate the 10:1 fixed-ratio of GT *w*/*w* in DcNP formulation against human breast cancer cells. As shown in Figure 8, we found that GT-in-DcNP treatment significantly inhibited MDA-231-HM human tumors in athymic mice, showing a 99.8% reduction in tumor growth compared to the saline-treated controls. In addition, time-course tumor growth analysis also revealed a trend toward human breast cancer growth suppression for tumors in axillary and some degree of regression in sub-iliac sites. However, the study is somewhat limited by the slow growth of the MDA-231-HM tumors in athymic mice requiring 28 days of study plan. Despite the limitation, the yet-to-be-optimized multiple-dosing schedule of GT-in-DcNP showed promising results in achieving human breast tumor suppression with the current fixed-dose ratio of the product. Collectively, these preliminary results provide early indications that the enhancement mediated by GT-in-DcNP may extend to human breast tumor interventions.

The DcNP technology was initially developed and demonstrated to stabilize hydrophobic and hydrophilic HIV drugs within lipid excipients. A simple, scalable manufacturing process was used to combine four different sets of HIV drug combinations in each nano-drug-combination in suspension [32]. This process has been successful in scaling up to and supporting large scale product preparation to support non-human primate studies. By combining chemically and physically diverse drugs together in a stable particle, DcNP is able to enhance the co-localization of drugs in target cells, enhance overall drug exposure, and maintain long-acting plasma drug kinetics [27,28,33,34]. GT-in-DcNP is an extension of that much larger body of work and represents an application of this technology toward a new therapeutic area, specifically metastatic cancer. With the successful formulation of GT-in-DcNP presented here, it is possible that DcNP technology can stabilize other combination regimens that are more potent or have a safer toxicity profile. Besides breast cancer combination regiments, we are also able to use DcNP technology to formulate two leukemia regiments: venetoclax (Bcl-2 target) and zanubrutinib (BTK target) as a synchronized and long-acting drug-combination for the treatment of leukemia [35]. A DcNP approach may represent a promising tool in the future to prevent tumor recurrence and distant metastasis, something that has not been achieved with current chemotherapy approaches.

Although G and T have been separately formulated into nanoparticles [36,37,38,39], investigations on GT co-formulation have been scarce. A few studies have reported results with a GT drug combination in nanoparticles for the treatment of metastatic breast cancer [40,41,42] with various degrees of formulation complexity and impact on tumor growth rates. For example, Zhang et al. have encapsulated GT (8:1 *w*/*w*) in asymmetric lipid layers to form sphere-like nanoparticles conjugated with cyclic arginylglycylaspartic acid (cRGD) peptide to enable tumor targeting. After three IV doses of GT (GT dose 16:2 mg/kg, every three days), tumors treated with nanoparticles were ~50% smaller than the free drug combination group, and the nanoparticles did not cause any systemic toxicity by day 8. However, the manufacturing process requires multiple preparation steps and harsh organic solvents, which are challenging for pharmaceutical product development [41]. Dong et al. co-formulated gemcitabine and paclitaxel (G:T 1:1 *w*/*w*) with methoxy poly (ethylene glycol)–poly(lactide-coglycolide) to form polymeric nanoparticle encapsulating GT (PG/PP). Mice subcutaneously implanted with 4T1 cells were used to evaluate the efficacy of GT nanoparticles on reducing the tumor size overtime. PG/PP (GT dose 0.25:0.25 mg/mouse) was administrated three times and monitored for 60 days. However, the efficacy from the PG/PP group was only slightly increased than the free drug combination group (~30%) [40]. In contrast, our GT-in-DcNP could provide ~100% tumor inhibition enhancement compared to the free drug combination (Figure 3). Furthermore, we demonstrated the ability of DcNP to deliver GT into tumors through preferential uptake and retention in the lymphatics (likely due to their unique morphology and an appropriate hydrodynamic size range [43,44]), which may play a crucial role in the early stage of the metastatic process [1,4,5]. As the GT-in-DcNP composition is intentionally designed to be simple and scalable without the need for free-drug removal, GT-in-DcNP is a promising candidate to consider as a long-acting, target-synchronized drug-combination product.

## 5. Conclusions

In summary, we have shown that the subcutaneous administration of GT-in-DcNP form significantly improves the inhibitory effects of GT combination therapy on metastatic breast cancer in an aggressive, multi-site primary tumor model. A single SC dose of GT-in-DcNP resulted in reducing tumor size at levels that cannot be achieved with freely soluble GT. It is likely that DcNP-mediated preferential lymphatic distribution and persistent accumulation and exposure of GT at or near the breast tumor have led to enhanced anti-tumor effects and tumor regression. The yet-to-be-optimized GT-in-DcNP effect on breast cancer was extended to a human xenograft orthotopic MDA-231-HM fat-pad tumor model. Overall, short-acting combination regimens such as GT used in metastatic breast cancer treatment can be transformed into targeted, long-acting drug-combination nanoparticles for synchronized tissue and cell-selective GT exposure enabled by the DcNP technology. In doing so, the DcNP platform may have the potential to design a curative treatment beyond GT drug combination for breast and other cancers in our effort to eliminate residual or advancing metastatic cells and thereby prevent tumor recurrence.

## Figures and Tables

**Table 1:** Effect of 4T1 cell number and injection volume on early primary tumor growth (days 1–5).

Cell Number	Volume	# of Tumors	Live Tumor Luminescence (Photon Counts) ^b^			Tumor Volume at Day 5 (mm^3^) ^a^
(Million)	Inoculant (µL)		Day 1	Day 3	Tumor Growth (Fold-Change)	
0.2	20	4	760 ± 894	7769 ± 6975	10.2	N/A ^d^
0.5	50	4	2617 ± 3294	14,759 ± 17,201	5.6	N/A ^d^
1	100 ^c^	4	4350 ± 995	23,435 ± 11,180	5.4	N/A ^d^
1	50	12	3244 ± 1942	16,021 ± 13,247	4.9	40 ± 17
1	50	16	1386 ± 1950	8389 ± 9889	6	53 ± 44

^a^ Tumors became palpable on day 5. ^b^ Baseline luminescence signal is 58 ± 39 in mice with no tumors (+luciferin). ^c^ 100 µL injection volume was too large for the mammary fat pads and resulted in excessive fluid buildup. ^d^ N/A, not applicable by caliper measurement. Data expressed were mean ± SD for all four (two at axillary and two at sub-iliac) inoculation sites.

## Data Availability

The data presented in this study are within the article and/or on request from the corresponding author.

## Supplementary Materials

The following supporting information can be downloaded at https://www.mdpi.com/article/10.3390/cancers16162792/s1. Figure S1. Effects of varying 4T1 cell numbers and volume on establishing an orthotopic tumor model, as determined by bioluminescence signal on day 3. Panel A: From right to left, mice were inoculated with 0.2 million cells in 20 µL, 0.5 million cells in 50 µL, and 1 million cells in 100 µL. Representative tumor luminescence images are presented for mice inoculated at four sites. Panel B: Replication of representative mice inoculated with 1 million 4T1 cells in 50 µL at four mammary fat pads. This cell number and volume are used for treatment and interventional studies described in the following studies. Table S1. Frequency and description of adverse events in mice treated subcutaneously with increasing doses of the gemcitabine and paclitaxel combination as GT-in-DcNP.

## Abbreviations

G	Gemcitabine
T	Paclitaxel
DcNP	Drug combination nanoparticles
IV	Intravenous/intravenously
SC	Subcutaneous/subcutaneously
DSPC	1,2-distearoyl-sn-glycero-3-phosphocholine
DSPE-mPEG_2000_	1,2-distearoyl-sn-glycero-3-phosphoethanolamine-N-[methoxy(polyethylene glycol)-2000]
ICG	Indocyanine green
GFP	Green fluorescence protein
IVIS	In vivo imaging system
IHC	Immunohistochemistry
AE%	Association efficiency
