# Supplementary material for: Drug Combination Nanoparticles Containing Gemcitabine and Paclitaxel Enable Orthotopic 4T1 Breast Tumor Regression
Source: Cancers (Basel). 2024 Aug 8;16(16):2792. doi: 10.3390/cancers16162792 (PMC11352501; doi:10.3390/cancers16162792)
Supplement: Supplementary file 1 [file cancers-16-02792-s001.zip › cancers-3050848-supplementary.pdf]

Supplementary information

# Drug Combination Nanoparticles Containing Gemcitabine and Paclitaxel Enable Orthotopic 4T1 Breast Tumor Regression

**Jesse Yu [1,†], Xiaolin Xu [1,†], James Ian Griffin [1], Qingxin Mu [1,*] and Rodney J. Y. Ho [1,2,*]**

[1] Department of Pharmaceutics, University of Washington, Seattle, WA 98195, USA; yuj73@uw.edu (J.Y.); alinxu@uw.edu (X.X.); jig4702@nyu.edu (J.I.G.)
[2] Department of Bioengineering, University of Washington, Seattle, WA 98195, USA
[*] Correspondence: qmu@uw.edu (Q.M.); rodneyho@uw.edu (R.J.Y.H.)
[†] These authors contributed equally to this work.

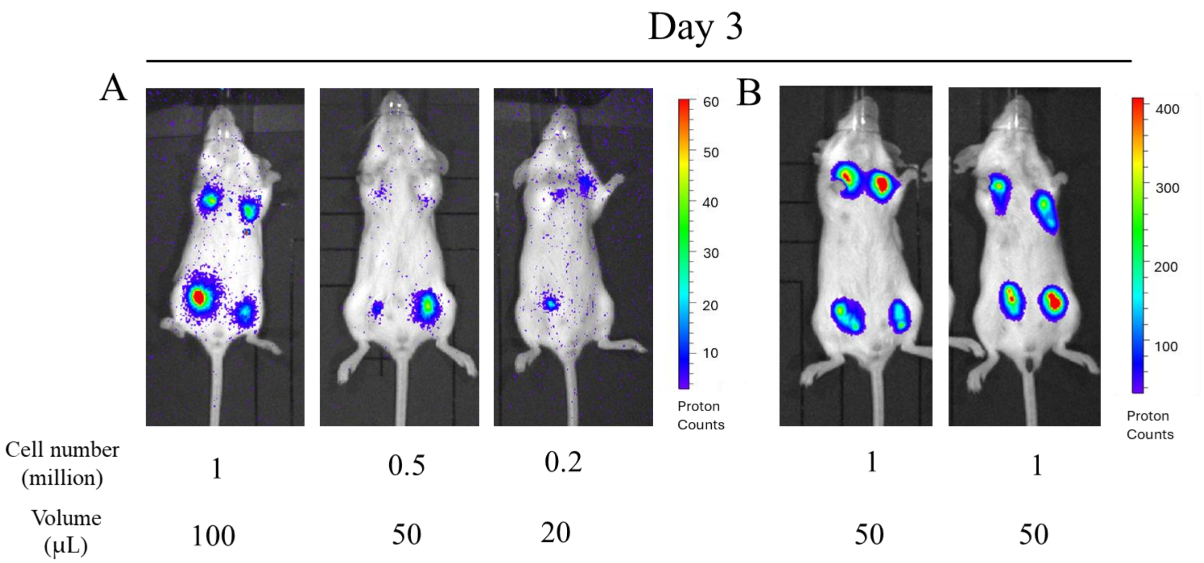


**Figure S1.** Effects of varying 4T1 cell numbers and volume on establishing an orthotopic tumor model, as determined by bioluminescence signal on day 3. Panel A: From right to left, mice were inoculated with 0.2 million cells in 20 μL, 0.5 million cells in 50 μL, and 1 million cells in 100 μL. Representative tumor luminescence images are presented for mice inoculated at four sites. Panel B: Replication of representative mice inoculated with 1 million 4T1 cells in 50 μL at four mammary fat pads. This cell number and volume are used for treatment and interventional studies described in the following studies.

**Table S1.** Frequency and description of adverse events in mice treated subcutaneously with increasing doses of the gemcitabine and paclitaxel combination as GT-in-DcNP.

| Gemcitabine: Paclitaxel dose (mg/kg) given as SC GT-in-DcNP | # of mice per group | # of mice with mild adverse event* | # of mice with significant adverse event** | Notes |
|---|---|---|---|---|
| 5 : 0.5 | 5 | 0 | 0 | No differences in behavior or health |
| 10 : 1 | 5 | 5 | 0 | Mild: injection site erythema, no changes in behavior |
| 20 : 2 | 5 | 4 | 1 | Mild: injection site erythema, no changes in behavior<br>Significant: anorexia and potential GI toxicity observed in necropsy |

*Mild adverse events: injection site reaction observed as redness and erythema within 7 days

**Significant adverse events: including >15% weight loss, anorexia, and GI toxicity observed at necropsy on day 7
